# Autophagic Regulation of p62 is Critical for Cancer Therapy

**DOI:** 10.3390/ijms19051405

**Published:** 2018-05-08

**Authors:** Md. Ariful Islam, Mopa Alina Sooro, Pinghu Zhang

**Affiliations:** 1 Jiangsu Key Laboratory of New Drug Screening & Jiangsu Center for Pharmacodynamics Research and Evaluation, China Pharmaceutical University, Nanjing 210009, China; ariful.islam1107@gmail.com (M.A.I.); mopa.sooro@yahoo.com (M.A.S.); 2 Institute of Translational Medicine & Jiangsu Key Laboratory of Integrated Traditional Chinese and Western Medicine for Prevention and Treatment of Senile Diseases, Medical College, Yangzhou University, Yangzhou 225001, China

**Keywords:** p62, PB1, self-oligomerization, autophagy, apoptosis, cancer, therapy

## Abstract

Sequestosome1 (p62/SQSTM 1) is a multidomain protein that interacts with the autophagy machinery as a key adaptor of target cargo. It interacts with phagophores through the LC3-interacting (LIR) domain and with the ubiquitinated protein aggregates through the ubiquitin-associated domain (UBA) domain. It sequesters the target cargo into inclusion bodies by its PB1 domain. This protein is further the central hub that interacts with several key signaling proteins. Emerging evidence implicates p62 in the induction of multiple cellular oncogenic transformations. Indeed, p62 upregulation and/or reduced degradation have been implicated in tumor formation, cancer promotion as well as in resistance to therapy. It has been established that the process of autophagy regulates the levels of p62. Autophagy-dependent apoptotic activity of p62 is recently being reported. It is evident that p62 plays a critical role in both autophagy and apoptosis. Therefore in this review we discuss the role of p62 in autophagy, apoptosis and cancer through its different domains and outline the importance of modulating cellular levels of p62 in cancer therapeutics.

## 1. Introduction

**Figure 1:**
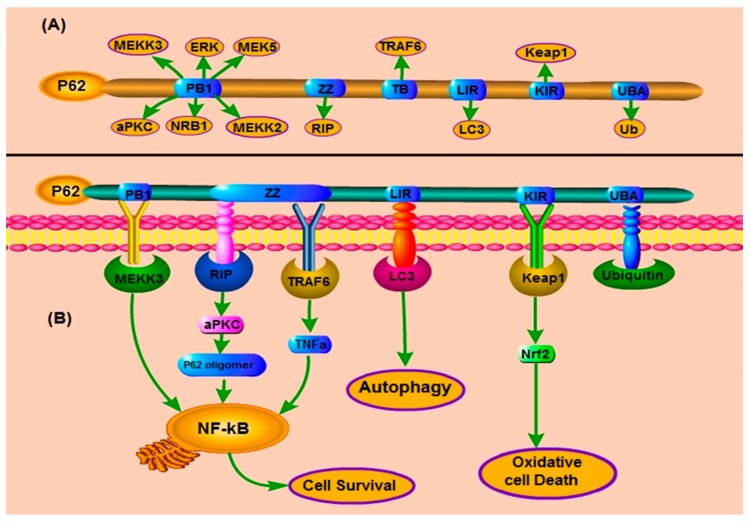
P62-interacting partners to activate different signaling pathways for cellular functions: (**A**) P62 has multiple domains, such as a PB1 domain, a ZZ-type zinc finger domain, a TRAF6-binding (TB) domain, an LC3-interacting region (LIR), KIR domain, and a ubiquitin-associated domain (UBA). PB1 domain plays an important role for self and hetero-oligomerization of p62 by binding with other PB1 containing protein, such as aPKC, ERK, and NBR1. P62 interacts with the RIP at ZZ zinc finger region, and TRAF6 at TB domain, which regulates NF-κB activation. P62 binds with LC3 by the LIR, and Keap1 by the KIR domain. The C-terminal UBA domain of p62 associates with ubiquitin; (**B**) P62 stimulates the oligomerization of aPKC and interaction with MEKK3 and caspase-8 leading to the enhancement of NF-κB activation. The LIR domain of p62 binds with LC3 to activate autophagy. Its KIR domain interacts through Keap1 to activate Nrf2 pathway that regulates oxidative stress.

P62, also well known as sequestosome 1 (SQSTM1) in humans, is a scaffold protein [1] and a stress-inducible protein [2] with multiple domains including a Phox1 and Bem1p (PB1) domain [3], tumor necrosis factor (TNF) associated receptor-6 (TRAF6) binding (TB) domain [4], a ZZ-type zinc finger (ZZ) Domain, an LC3-interacting region (LIR), a Keap1-interacting region (KIR), an ubiquitin-associated domain (UBA) (Figure 1A) [5]. These multiple domains have been indicated central to p62’s various functions in processes such as autophagy [6], apoptosis, inflammation [1], cell survival [7], cell death [8], signal transduction [9], and tumorigenesis [10]. P62 is further a mono or polyubiquitin-binding protein that accumulates in different chronic, toxic, and degenerative diseases due to aggregation of the cytosolic protein [11]. Through regulation of autophagy and apoptosis, this multifunctional protein and signaling hub functions to control cell viability in response to cytotoxic stress [12]. This protein thus plays an important role in cancer. P62 is an autophagy receptor and a selective substrate for autophagy [13]. Autophagy is the main pathway by which the cell clears harmful materials from the cytoplasm and the autophagosomes deliver this target cargo to the lysosomal system for degradation [14]. P62 can directly interact with LC3 for autophagosome formation [15]. The protein’s degradation is dependent on autophagy [16]. P62 is further important for the induction of apoptosis, for example through the caspase-8 activation at the autophagosomal membrane i.e., the autophagy-dependent mechanism of caspase-8 activation [17]. Consequently, our review will discuss how autophagic regulation of p62 is critical for cancer therapy through its multiple domains. The relationship between p62 and various known autophagy and apoptosis-regulating proteins will be elaborated. The potential of p62 manipulation for cancer therapeutics will thereby be unveiled.

## 2. The Role and Function of p62/ SQSTM1 through Its Different Domains

### 2.1. The Role of PB1 Domain of p62

It has been established that one of six domains that p62 contains is an N-terminal Phox/Bem1p (PB1) domain, which mediates the efficient formation of p62 polymers, where the acidic surface residue Asp 69 (D69) of one p62 PB1 domain binds to a basic surface residue Lys 7 (K7) in the next PB1 domain by hydrogen bonding [16]. The PB1 domain of p62 has been reported to be critical for its various defined cellular and physiological functions. A number of proteins containing the PB1 domain have been discovered and demonstrated to interact with each other through this domain especially through their basic front surface [18]. For example, Lamark et al. have revealed critical interactions between atypical protein kinase C (aPKC) and p62 which are crucial for recruitment of the aPKC into the tumor necrosis factor-α and interleukin-1 receptor signaling complexes [3]. Indeed, p62 through its PB1 domain regulates the activity of PKCζ and by its binding to this protein activates the PKCζ-JNK-caspase 3 apoptotic pathway in endothelial cells [19]. Another recently unveiled regulatory mechanism of this multi-domain signaling hub is the cyclic AMP-dependent protein kinase signaling. P62 couples with the cAMP signaling system by direct binding of cAMP-degrading phosphodiesterase-4 (PDE4) to the acidic surface of the p62 PB1 domain and phosphorylation of the basic surface of this domain by cAMP-dependent protein kinase (PKA). This disrupts the interaction of the PB1 domain with other binding partners.

MEK5 has been reported as the only MAP2K that expresses the PB1 domain. Nakamura K and colleagues have thereby further demonstrated the functional form of a MEKK2-MEK5-ERK5 complex through their PB1 domains [20]. Linares et al. discovered a kinase cascade in which the PBI-containing kinase MEKK3 interacted with the p62 PB1 domain and controlled the activity of mTORC1. When there is abundance of nutrients, such as amino acids, the MEKK3 phosphorylated MEK3/6 which in turn phosphorylated p38δ which further phosphorylates p62, thereby recruiting the TRAF6 to p62 and promoting mTOR translocation to the lysosome [21]. Furthermore, the mutational analysis performed in the study by Qiong group have proven that NEDD4 (neural precursor cell expressed developmentally down-regulated protein 4) interacts with and ubiquitinates the p62 PB1 domain. This domain is therefore an important factor for NEDD4 function as an autophagic E2 ubiquitin ligase that ubiquitinates p62 and facilitates p62-mediated inclusion body autophagy [22].

The ERK5 pathway plays a crucial role in cardiac development [23], angiogenesis [24], neuron survival [25] and cytoprotection against stress-induced apoptosis [26]. In hepatocytes, hepatitis B virus (HBV) X protein (HBx) stimulates the expression of Glucose-6-phosphate dehydrogenase (G6PD) in an NF-E2-related factor 2 (NRF2) activation-dependent pathways. The HBx makes this association with the UBA and PB1 domains of p62. This proves the impact of HBV on the reprogramming of the glucose metabolism in hepatocytes, crucial in the development of HBV-associated hepatocarcinoma [27]. The neighbor of BRCA1 gene 1 (NBR1) protein in this domain has also been demonstrated to bind with p62 [20]. NBR1 and p62 co-operate in the sequestration of misfolded and ubiquitinated proteins for degradation by the lysosomal system during autophagy [3]. In impaired autophagic flux, Lee et al. have demonstrated that p62 is polyubiquitinated to prevent its oligomerization [28]. However, Cohen-Kaplan et al. have discovered that deletion of the p62 PB1 domain has no effect on stress-induced autophagy of the lysosome degradation machinery. This suggests that PB1 domain of p62 might play a critical role in determining the function of p62 targeting substrates to the proteasome or targeting to autophagy lysosomal system [29]. Most recently, p62 has been shown to promote tumorigenesis in autophagy-deficient tumor cells by altering NF-κB regulation through its PB1 domain [20]. Together, these results prove that PB1 domain is significant for protein-protein interaction leading to p62 oligomerization.

### 2.2. The Role of ZZ Domain of p62

The ZZ zinc finger domain of p62 is known to be the binding site for RING finger protein tumor necrosis factor (TNF) receptor-associated factor 6 (TRAF6). It is also responsible for binding to receptor-interacting protein (RIP), a TNFα signaling adaptor (Figure 1B) [30].

For example, Yu and colleagues showed that this complex can also activate the NF-ĸB and p38MAPK signaling pathway. Using a combined stable isotope labeling with amino acid in cell culture proteomics methodology, it was indeed observed that the p62 ZZ-type zinc finger domain is the innate defense regulator 1 (IDR-1) binding site. Therefore it is a potential therapeutic target for inflammatory and infectious diseases [31]. Sanz et al. demonstrated that p62 can interact with RIP and link the aPKCs to activate NF-κB involving the TNF-R1/TRADD/RIP/p62/aPKCs/IKKβ signaling pathway [30]. In addition, p62 is also a significant intermediate in an IL-1 signaling pathway to activate NF-κB through the specific adapters such as RIP and TRAF6 (Figure 1A) [18]. In cisplatin-resistant ovarian (SKOV3/DDP) cells, loss of the p62 ZZ domain restored sensitivity to cisplatin treatment. It was proven that this domain activity inhibited apoptosis thus mediating drug resistance in ovarian cancer cells [32]. A novel p62-ZZ inhibitor XRK3F2 is involved in inhibiting multiple myeloma (MM) cell growth, as well as BMSC growth enhancement of human MM cells [33]. Further evidence indicated that XRK3F2 has a relative specificity for p62-ZZ and characterized XRK3F2’s capacity to inhibit the growth of primary MM cells and human MM cell lines [34]. Moreover, the p62 ZZ domain aids the degradation of misfolded protein through autophagy, by its interaction with the N-terminal Arginine of ArginyatedBiP (R-BiP). This interaction induces p62 self-oligomerization and aggregation which increases its association with LC3 thereby selective lysosomal degradation of target cargo as well as these proteins [18,30]. Furthermore, p62 is also an AMPA receptor interacting protein (RIP). Mechanistic studies demonstrated that there is an interaction between p62 and AMPA receptor-mediated through the AMPA receptor subunit intracellular loop L2-3 and the ZZ domain of p62 [35]. These results proposed that ZZ zinc finger domain of p62 is significant for self-oligomerization of p62, which can activate the NF-κB signaling pathway and result in selective degradation of p62.

### 2.3. The Role of LIR Domain of p62

LC3-interacting region (LIR) is required for the autophagy degradation of the mammalian protein [36]. P62 interacts with LC3 through LIR domain to promote the formation of autophagosome [13]. In addition, p62 can also interact with other autophagic effector proteins like as LC3A, LC3B, beclin-1, BNIP3 [37], gamma-aminobutyric acid receptor-associated protein (GABARAP) and GABARAP-like molecules through LIR domain [11]. These proteins are involved in autophagic degradation of cargos in the selective way that is ensured by binding of this region of the receptor protein p62 with Atg8/LC3/GABARAPs protein. This domain thus link the core autophagy machinery to the target cargo [38]. Furthermore, knockdown of LC3B results in a significant accumulation of selective substrate p62 [39]. It is, therefore, evident that p62 as an autophagy receptor plays a critical role in the selective autophagosome degradation of its ubiquitinated target proteins [40]. Identification and characterization of LIR-containing proteins such as ULK1, ATG13, FIP200, and Dvl2 interaction with LC3 give significant insights in the study of the autophagy pathway. It represents a current rising area of autophagy research [38]. There is recently developed new sensor; HyD-LIR-GFP which by using LIR motif from FYCO1, specifically detects LC3A/B-positive localization at autophagosome in the presence of endogenous LC3/GABARAP to Mito Tracker-positive damaged mitochondria upon mitophagy induction. It is an excellent novel autophagosome sensor in autophagy for bulk degradation of cytosolic components in lysosomes [41]. The LIR mutation is required to impair Spred2-mediated tumor cell death in an autophagy-dependent manner [42]. Taken together, these evidences indicate that LIR-interacting region of p62 is a key domain for regulating the autophagic degradation of selective ubiquitinated proteins in mammalian cells.

### 2.4. The Role of KIR Domain of p62

The KIR domain of p62 is located next to the LIR domain and resembles the ETGE motif utilized by Nrf2 for its interaction with Keap1 [43]. Under normal conditions, Keap1 negatively regulates the expression of Nrf2 by promoting its ubiquitination, resulting in the rapid degradation of Nrf2 by the ubiquitin-proteasome system [44,45]. However, under abnormal conditions, such as oxidative stress, p62 can also compete with Nrf2 for binding to Keap1. Furthermore, p62 binds to Keap1for dissociation of Nrf2 from Keap1 to promote Nrf2 translocation to the nucleus, inducing the transcription of detoxification enzymes and oxidative stress response genes [43,46,47]. For example, sulforaphane is a potent inducer of the Keap1-Nrf2 signaling pathway which has a critical function to regulate cell defense against oxidative stress and to control the cellular redox balance at physiological condition [48]. The residues 349-DPSTGE-354 in p62 and three arginines in KIR are required for the direct interaction between p62 and Keap1 [47]. Thus, this interaction can promote Keap1 accumulation in p62 bodies leading to autophagic degradation of Keap1 [43]. Moreover, p62 acts as an adaptor protein to increase Nrf2 activation by the impairment of Keap1 activity in the autophagic process [49]. In addition, KIR is required for p62 to stabilize Nrf2 activation through suppression of Keap1 in response to oxidative stress [43]. Taken together, these results prove that KIR domain of p62 can regulate the accumulation of Keap1 for autophagic degradation to activate Nrf2 during induction of oxidative stress for the positive feedback loop from a cytoplasmic location in cells.

### 2.5. The Role of UBA Domain of p62

The UBA domain of p62 has a significant role in the function of p62 [29]. UBA domain-mediated p62 proteasome interaction plays a role in the targeting of the proteasome and the autophagy-lysosome system. UBA domain of p62 is a core regulator of the interaction between p62 and the ubiquitinated proteasome (Figure 1B) [50]. The p62 protein can regulate the interaction between ubiquitinated proteins and autophagosome through its UBA domain, where autophagosome is a central controller for this protein degradation via the autophagy-lysosomal pathway [29]. The earlier results strongly suggest that after amino acid starvation, p62 recognizes the ubiquitinated proteasome and does this through its association with LC3B, and thereby facilitating autophagosomal recognition of the proteasome. In general, this is the case in the recognition and damage of other cargoes as well [11]. Importantly, depletion of endogenous p62 can reduce the proteasome after starvation, again highlighting the core role of p62 in the recruitment of ubiquitinated proteasomes into autophagosomes [29]. The importance of the UBA domain of p62 has also been emphasized when it was found that the mutations of the UBA domain are frequently associated with familial and sporadic Paget’s disease of bone. In other words, mutation of UBA domain activates TRAF6–NF-κB signaling which result is increased osteoclastogenesis [6,51,52]. It is characterized by increased bone turnover which results in choric and metabolic disorder [52,53,54]. There is vastly known that RANKL and its receptor RANK play an important role in regulating osteoclast differentiation, activity, and survival by activating NF-κB [55]. It has been proposed that p62 enables to create a complex form between TRAF-6 and aPKC, which form is RANKL-induced in NF-κB activation [4,55,56]. P62 KO mice have a similar phenotype of Paget’s disease with impaired of NF-κB activation and also supporting that p62 is an important to promote bone homeostasis [9,55]. Taken together, these results suggested that UBA domain of p62 is crucial for interaction between ubiquitinated protein and autophagosome.

## 3. P62 and Autophagy

**Figure 2:**
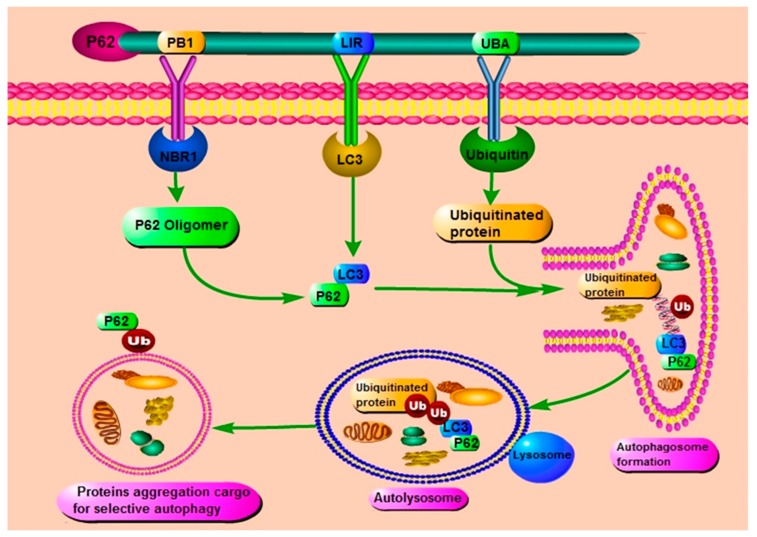
Control of p62 by autophagy: P62 and NBR1 are bound together by their PB1 domains to induce p62 oligomerization that allows binding to LC3 to form p62-LC3 through the LIR domain. P62 by its LIR domain interacts with the LC3 to connect cargo to the autophagy machinery and UBA domain binds with ubiquitinated protein by ubiquitin. These protein complexes can regulate autophagosome formation to regulate proteins aggregation cargo for selective autophagy.

There is currently a general consensus that p62 is an effector of selective autophagy as well as a substrate to autophagy [2]. Autophagy is a programmed degradation of cytotoxic components through the lysosomal system [57]. The p62 protein through its LIR domain interacts with LC3 for attachment to the autophagosomes, thereby delivering the ubiquitinated target cytotoxic materials attached to it through its UBA domain for transportation to the lysosome and degradation (Figure 2) [11,50]. At physiological conditions, basal autophagy functions as a signal transduction adaptor and the levels of p62 are relatively low due to continuous degradation by autophagy [58]. P62 is a well-known significant regulator of selective autophagy, functioning in cargo formation of the autophagic machinery [59]. For instance, it can bind with the NBR1 through its PB1 domains and this results in the clearance of polyubiquitin, protein aggregates, misfolded, and dysfunctional organelles in mammalian cells by the autophagic machinery [11,40,60]. Basal autophagy maintains the levels of p62 at relatively low levels as it is degraded together with the target cargo by the lysosomal system. Indeed, maintenance of p62 levels at physiological levels through active autophagy strongly correlate with better control of diseases and prevents the development of cancers [58]. P62 transfers ubiquitinated protein aggregation to the autophagy-lysosome pathway during autophagy [12]. Autophagy receptor p62 can directly bind to ubiquitinated cargoes by its UBA domain and deliver them to phagophores via its PB1 and LIR domain [61]. Keap1/Cullin3 ubiquitination of p62 at lysine 420 by its UBA domain may regulate the function of p62 to disrupt in p62-associated diseases [62]. The multifunctional protein p62 interacts with Atg8/LC3 through the LC3-interaction region (LIR) and there is the accumulation of p62 in autophagy-deficient mice. As a result, studies in autophagy-deficient mice have exposed the molecular mechanism linking between autophagy and p62 [63]. Itakura et al. have reported that p62 is essential for its localization to autophagosome formation sites through interaction with LC3 on the endoplasmic reticulum (ER) [64]. P62 is an autophagic receptor protein that is associated with ER for autophagic degradation of excess hepatic ER after withdrawal of 1,4-bis[2-(3,5-dichloropyridyloxy)] benzene (TCPOBOP) in liver-specific Atg5 and p62 knockout mice [65]. Autophagic protein Atg5 is significant for inhibition of autophagy. P62-dependent hepatic differentiation is activated by autophagy inhibition. Autophagy inhibition can cause p62 accumulation when there are high levels of amino acid and mTOR pathway activated. mTOR pathway is increased by Atg5 silencing and suppressed by p62 silencing [66]. Furthermore, Atg3, Agt4B, Atg7 and beclin1 protein in the liver are also associated with a decreased LC3 and the accumulation of p62 by chronic activation of PPARα agonist fenofibrate (FB) to autophagic degradation [67]. LC3 is most reliable autophagy marker which can be accumulated that was observed of neurite degeneration by knocking down of Atg7 and Beclin1 after nerve growth factor deprivation [68]. Autophagy is strongly induced by suppressing mTOR kinase activity during nutrient deprivation. It activates cathepsin-positive autolysosome than LC3II-positive autophagosome thereby resulting in fusion with lysosome and degradation of healthy neurons. It is suggested that this process is the main pathway for organelle and protein turnover in neurodegeneration of Alzheimer’s disease (AD) [69]. LC3 is the processing protein in the spinal cord and activates motor neurons (MNs) of Zn-superoxide dismutase (SOD1) mice, suggesting a potential role of autophagy in the pathogenesis of amyotrophic lateral sclerosis (ALS) [70]. In contrast, this recent evidence indicates that the absence of Atg8 family LC3/GABARAP proteins could not prevent the formation of autophagosome during PINK1-PARK2-dependent mitophagy. However, this protein is important for autophagosome size and stimulates autolysosome formation in autophagy [71]. Autophagy is a selective degradative process which requires various autophagic receptor proteins. These can selectively recognize ubiquitinated cargoes and deliver them to phagophores, which is a pioneer to autophagosomes for autophagic degradation (Figure 2). P62 is a core regulator to bind to ubiquitinated protein aggregates and thereby processes them into inclusion bodies [61,62].

P62 and NBR1 are bound by their PB1 domain to induce p62 oligomerization that allows binding to LC3 to form p62-LC3 through LIR domain of p62. LIR domain of p62 induces autophagy by binding with LC3 and UBA domain binds with ubiquitinated protein by ubiquitin. These binding can regulate autophagosome formation, consequently which processes autolysosome by the presence of lysosome to regulate proteins aggregation cargo for selective autophagy.

Oligomerization of p62 serves as a new indication of the exposure of LC3-binding proteins to autophagic degradation of ubiquitinated diseases-related protein aggregation for liver diseases and neurodegenerative diseases such as Alzheimer’s disease (AD), Parkinson’s disease (PD) [7,16,72]. In the past decade, it has been demonstrated that p62 is receptor protein by interaction with LC3 to ensure the cargo selectivity for autophagy degradation. There is core autophagy machinery to target the protein, an organelle, and pathogen [38]. There is high accumulation of p62 together with polyubiquitinated proteins aggregates in various chronic, toxic, and degenerative diseases [11]. These results suggest that p62 has a vital role as a receptor protein for cargo selectivity by autophagy degradation.

## 4. P62 and Cancer

Cancer arises from uncontrolled proliferation and sustained defective cell survival with a potential to invade or spread to other parts of the body [73]. The upregulation and/or inefficient degradation of p62 has been linked with tumorigenesis [74]. The sustained p62 expression resulting from autophagy defects was observed sufficiently to alter NF-κB regulation and gene expression thereby promoting the formation of a tumor [43]. Takamura et al. has reported that growth of liver tumors caused by the inhibition of autophagy is greatly diminished by concomitant deletion of p62 or Nrf2 [75]. Abnormally accumulated inclusions were observed in human hepatocellular cancer tissue in which p62 was discovered to be the major component of these inclusions [76].

The rise in p62 levels due to its elevated transcription or low levels of autophagy has further been demonstrated in oncogenesis and resistance to cancer therapy as well as in numerous other diseases. Tumor promotion activity of p62 was observed in hepatitis B virus (HBV)-associated hepatocarcinoma. HBV is involved in the reprogramming of the glucose metabolism in hepatocytes leading to hepatocarcinoma [27]. This is the case even in breast [77], gastric [78] and prostate cancers [79]. In human liver diseases like non-alcoholic steatohepatitis (NASH) and hepatocellular carcinoma (HCC), p62 was frequently observed to be accumulated [76]. Intracytoplasmic hyaline in cancer cell has mallaorybodie (MB)-type and hyaline globule (HG)-type inclusions. These have high levels of p62 and ubiquitin. Cholangiocarcinomas are promoted by these hyaline inclusions. This result implies that intracytoplasmic hyaline bodies are often found in cholangiocarcinoma in chronic liver disease related to alcohol intake and viral infection such as HBV or HCV [80].

Not only are the high levels of p62 been observed in tumor promotion but they have strongly further been implicated in causing resistance to cancer therapy especially platinum-based therapeutic strategies [32]. Over-expression of p62 was observed in cisplatin-resistant ovarian epithelial carcinoma and reduction of the p62 levels through autophagy upregulation increased the tumor sensitivity to the drug [81]. Indeed increased levels of p62 may indicate insufficient autophagy for p62 degradation and this also leads to resistance to apoptosis [82]. In esophageal squamous cell carcinoma (ESCC) tissues p62 levels were found to be upregulated and demonstrated to be the main regulator of cells apoptosis both in vitro and in vitro. Through activation of protein kinase C iota (PKCiota)-S phase kinase-associated protein 2 (SKP2) signaling pathway, p62 enhanced cell apoptosis resistance and thus promoted tumor growth [83]. It has been established that the tumor microenvironment is an indispensable factor for consideration in cancer therapy, therefore p62 activity in the microenvironment is important to observe. P62 has been reported to be an anti-inflammatory tumor suppressor through its regulation of the mTORC1/c-Myc-pathway of stromal glucose and amino acid metabolism in epithelial prostate cancer cells [84]. Furthermore, this protein has been found to control DNA-damage-induced histone H2A ubiquitination in autophagy-deficient cells. High levels of p62 were proven to inhibit E3 ligase RNF168 activity which is crucial for H2A ubiquitination, thereby inhibiting DNA-damage repair mechanism and thus increases sensitivity of cancer cells to radiation [85,86]. In these cases, p62 upregulation was observed to be advantageous for cancer therapy. However, the high frequency of tumor-initiation in high p62 expressing cancer cells is an important factor that cannot be denied [87]. Therefore, Leidal and Debnath have proposed the current need to inhibit both autophagy and p62 if autophagy inhibition strategy against cancers is to be successful [88].

Various studies have proven the activity of cancer stem cells (CSCs) within tumours. CSCs are implicated to be the cause for most cancers therapeutic resistance, relapse and metastasis. Indeed, in breast cancer cells, there was an additional layer of heterogeneity that was proven to be behind the self-renewing tendencies of the cancer cells. P62 was further demonstrated to be highly expressed in this layer and its depletion by siRNA robustly inhibited this resistance in both in vivo and in vitro studies [87,89]. This therefore indicates the potential of p62 as a therapeutic target for most cancers with cancer stem cells.

The human cholangiocarcinoma cell line with cisplatin resistance was used to investigate the role of autophagy in chemotherapeutic drug resistance. The pentose phosphate (PPP) pathway activity was demonstrated to be promoted more after cisplatin treatment and this pathway is observed to be the one behind drug resistance in cholangiocarcinoma cells. The detected p62 and LC3 autophagy substrates proved a higher flow of autophagy in cholangiocarcinoma cells [90]. Autophagy inhibitor chloroquine can significantly increase the sensitivity of cells to cisplatin by blocking autophagy-lysosome pathway of cholangiocarcinoma which may be a new route for therapeutic agents against cholangiocarcinoma [90]. The combination of ABT737 with cisplatin promotes cholangiocarcinoma cell death by apoptosis. This combination increases sensitivity to cisplatin by controlling of the mitochondrial dynamics. The mitochondrial dynamics play a significant function in the response of cholangiocarcinoma to cisplatin [91]. Zhang et al. demonstrated that oblongifolin C (OC) can decrease cell viability and induce apoptosis in a dose dependent manner through inhibition of autophagy and mitochondrial dysfunction (MtD) [92]. Further evidence indicates that in cholangiocarcinoma cells, FOXO1 is involved in the regulation of autophagy, oxidative stress, and MtD and it lowers the autophagy flux. Hence, it can be a potential therapeutic target for this cancer [93].

## 5. P62 and Apoptosis

**Figure 3:**
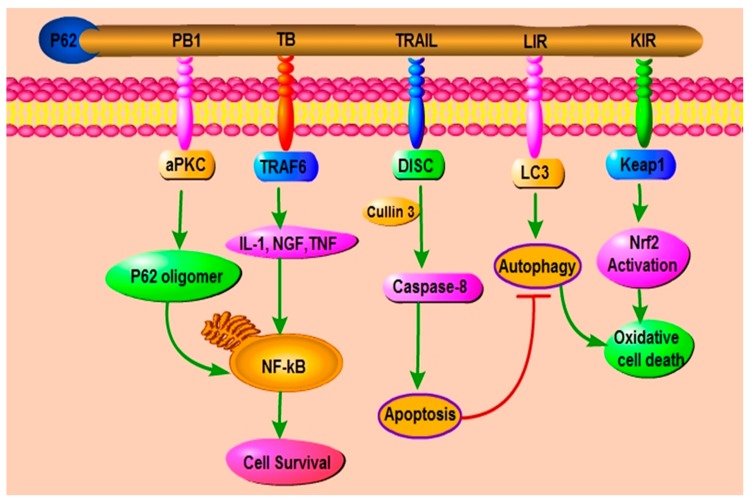
P62 mediate cell survival, cell death, and apoptosis: P62 stimulates the oligomerization of aPKC and TRAF6 binding TB domain leading to the enhancement of NF-κB activation to promote cell survival. P62 may activate caspase-8 by the presence of cullin3 which can induce apoptosis by autophagy inhibition. Caspase-8 activation and p62 oligomerization promote cell apoptosis.

Apoptosis is a well-known mechanism of programmed cell death (PCD) and it is mainly induced by activation of a family of cysteine proteases known as caspases. Apoptosis commitment requires efficient activation and autocatalytic release of caspase-8 into cytoplasm to engage executioner caspases [94]. There is recently strong evidence of the correlation between p62 and apoptosis. For example, p62 was observed in cytosolic aggregates formed of PB1-driven p62 oligomers and p62-aPKC. These were recognized as signal-organizing centers where p62 interacted with caspase-8 and TRAF6 thereby activating the caspase-8 downstream effectors caspases [18,94,95,96]. Therefore, this interaction triggered the apoptotic pathway [94]. In their study on the mechanism of full activation and stimulation of caspase 8, Jin Z et al. discovered that p62 promoted aggregation of CUL3-modified caspase-8 within p62-dependent foci (Figure 3), thereby leading to full activation and processing of the enzyme and full commitment to cell death [94].

We have previously demonstrated that among the most efficient cancer therapeutic strategies, especially against gastric cancer cells, are those that use the autophagy-dependent apoptosis to induce cell death [57,97]. Several cases of autophagy-dependent apoptosis with the identified involvement of p62 have also been reported. For example, in cisplatin-resistant ovarian (SKOV3/DDP) cells the levels of p62 were observed to be relatively high. The loss of the p62 ZZ domain restored sensitivity of the cells to apoptotic death [32]. Furthermore, the anti-apoptotic role of p62 causes activation of the p62-PKCiota-SKP2 pathway in esophageal squamous cell carcinoma (ESCC) through stabilizing SKP2 under serum starvation condition [83]. Recent studies demonstrated that K63-linked ubiquitination of RIP1 and the activation of the NF-κB signaling was higher in cisplatin-resistant ovarian cells rather than parenteral cells. The activation of the NF-κB signaling pathway is mediated by p62 and is partly dependent on RIP1. P62 stimulates cell proliferation and inhibits apoptosis thereby causing drug resistance in SKOV3/DDP cells [32]. In contrast, Galectin-3 (Gal-3) expression level was not affected by the inhibition of the NF-κB pathway in ovarian cancer cells. It may affect the migratory and invasive capabilities of cancer cells. These significantly promoted carcinogenesis while apoptosis and the cells’ sensitivity to carboplatin decreased. Epithelial ovarian cancer (EOC) become sensitive to carboplatin through induction of the NF-κB pathway [98]. Development of chemoresistant is a key barrier to successful treatment of patients affected by epithelial ovarian carcinoma. This studies suggested that NF-κB signaling is significant in TLR-4/MyD88 deficient receptor for the development of cisplatin resistance as it mediates the cell survival pathway in epithelial ovarian carcinoma [99]. It is indeed evident that p62 acts as a ubiquitinated cargo and a signaling hub to control cell survival, apoptosis, and inhibition of tumorigenesis by autophagy.

## 6. Concluding Remarks and Future Direction

It is apparent that p62 is one protein that cannot by any means be neglected when designing cancer therapeutic strategies. Recently there have emerged numerous studies suggesting a combination of autophagy inhibitors with epidermal growth factor receptor tyrosine kinase inhibitors (EGFR-TKIs) in cancers resistant to the latter therapy. These cancers were discovered to upregulate autophagy as a cytoprotective means. However, with this evidence that autophagy-deficient cells accumulate p62, thereby posing another tumorigenic potential, this combinatory strategy may not be the best for most cancers. The possibility of inhibition of both autophagy and p62 has indeed been strongly suggested. We therefore further press on the immediate need for in-depth research on p62 inhibitors if the current autophagy inhibition strategies under clinical trials are to be more successful. Further studies on the pathways that p62 modulate to effect its tumorigenic effects needs to be undertaken as well as studies that shed more insight into the protein’s function in physiologically relevant models.

## References

[1] Christian F., Krause E., Houslay M.D., Baillie G.S. (2014). PKA phosphorylation of p62/SQSTM1 regulates PB1 domain interaction partner binding. Biochim. Biophys. Acta.

[2] Katsuragi Y., Ichimura Y., Komatsu M. (2015). p62/SQSTM1 functions as a signaling hub and an autophagy adaptor. FEBS J..

[3] Lamark T., Perander M., Outzen H., Kristiansen K., Øvervatn A., Michaelsen E., Bjørkøy G., Johansen T. (2003). Interaction codes within the family of mammalian Phox and Bem1p domain-containing proteins. J. Biol. Chem..

[4] Wooten M.W., Geetha T., Seibenhener M.L., Babu J.R., Diaz-Meco M.T., Moscat J. (2005). The p62 scaffold regulates nerve growth factor-induced NF-κB activation by influencing TRAF6 polyubiquitination. J. Biol. Chem..

[5] Lin X., Li S., Zhao Y., Ma X., Zhang K., He X., Wang Z. (2013). Interaction domains of p62: a bridge between p62 and selective autophagy. DNA Cell Biol..

[6] Chamoux E., McManus S., Laberge G., Bisson M., Roux S. (2013). Involvement of kinase PKC-zeta in the p62/p62 P392L-driven activation of NF-κB in human osteoclasts. Biochim. Biophys. Acta.

[7] Bjørkøy G., Lamark T., Brech A., Outzen H., Perander M., Øvervatn A., Stenmark H., Johansen T. (2005). p62/SQSTM1 forms protein aggregates degraded by autophagy and has a protective effect on huntingtin-induced cell death. J. Cell Biol..

[8] Schläfli A.M., Adams O., Galván J.A., Gugger M., Savic S., Bubendorf L., Schmid R.A., Becker K.-F., Tschan M.P., Langer R. (2016). Prognostic value of the autophagy markers LC3 and p62/SQSTM1 in early-stage non-small cell lung cancer. Oncotarget.

[9] Manley S., Williams J.A., Ding W.-X. (2013). Role of p62/SQSTM1 in liver physiology and pathogenesis. Exp. Biol. Med..

[10] Galvez A.S., Duran A., Linares J.F., Pathrose P., Castilla E.A., Abu-Baker S., Leitges M., Diaz-Meco M.T., Moscat J. (2009). Protein kinase Cζ represses the interleukin-6 promoter and impairs tumorigenesis in vivo. Mol. Cell. Biol..

[11] Pankiv S., Clausen T.H., Lamark T., Brech A., Bruun J.-A., Outzen H., Øvervatn A., Bjørkøy G., Johansen T. (2007). p62/SQSTM1 binds directly to Atg8/LC3 to facilitate degradation of ubiquitinated protein aggregates by autophagy. J. Biol. Chem..

[12] Huang S., Okamoto K., Yu C., Sinicrope F.A. (2013). p62/sequestosome-1 up-regulation promotes ABT-263-induced caspase-8 aggregation/activation on the autophagosome. J. Biol. Chem..

[13] Ichimura Y., Kominami E., Tanaka K., Komatsu M. (2008). Selective turnover of p62/A170/SQSTM1 by autophagy. Autophagy.

[14] Wurzer B., Zaffagnini G., Fracchiolla D., Turco E., Abert C., Romanov J., Martens S. (2015). Oligomerization of p62 allows for selection of ubiquitinated cargo and isolation membrane during selective autophagy. eLife.

[15] Komatsu M. (2011). Potential role of p62 in tumor development. Autophagy.

[16] Bjørkøy G., Lamark T., Pankiv S., Øvervatn A., Brech A., Johansen T. (2009). Monitoring autophagic degradation of p62/SQSTM1. Methods Enzymol..

[17] Young M.M., Takahashi Y., Khan O., Park S., Hori T., Yun J., Sharma A.K., Amin S., Hu C.-D., Zhang J. (2012). Autophagosomal membrane serves as platform for intracellular death-inducing signaling complex (iDISC)-mediated caspase-8 activation and apoptosis. J. Biol. Chem..

[18] Sanz L., Diaz-Meco M.T., Nakano H., Moscat J. (2000). The atypical PKC-interacting protein p62 channels NF-κB activation by the IL-1–TRAF6 pathway. EMBO J..

[19] Kim G.-Y., Nigro P., Fujiwara K., Abe J.-I., Berk B.C. (2012). p62 binding to protein kinase C ζ regulates tumor necrosis factor α–induced apoptotic pathway in endothelial cells. Arterioscler. Thromb. Vasc. Biol..

[20] Nakamura K., Kimple A.J., Siderovski D.P., Johnson G.L. (2010). PB1 domain interaction of p62/sequestosome 1 and MEKK3 regulates NF-κB activation. J. Biol. Chem..

[21] Linares J.F., Duran A., Reina-Campos M., Aza-Blanc P., Campos A., Moscat J., Diaz-Meco M.T. (2015). Amino acid activation of mTORC1 by a PB1-domain-driven kinase complex cascade. Cell Rep..

[22] Lin Q., Dai Q., Meng H., Sun A., Wei J., Peng K., Childress C., Chen M., Shao G., Yang W. (2017). The HECT E3 ubiquitin ligase NEDD4 interacts with and ubiquitylates SQSTM1 for inclusion body autophagy. J. Cell Sci..

[23] Regan C.P., Li W., Boucher D.M., Spatz S., Su M.S., Kuida K. (2002). Erk5 null mice display multiple extraembryonic vascular and embryonic cardiovascular defects. Proc. Natl. Acad. Sci. USA.

[24] Sohn S.J., Sarvis B.K., Cado D., Winoto A. (2002). ERK5 MAPK regulates embryonic angiogenesis and acts as a hypoxia-sensitive repressor of vascular endothelial growth factor expression. J. Biol. Chem..

[25] Watson F.L., Heerssen H.M., Bhattacharyya A., Klesse L., Lin M.Z., Segal R.A. (2001). Neurotrophins use the Erk5 pathway to mediate a retrograde survival response. Nat. Neurosci..

[26] Kesavan K., Lobel-Rice K., Sun W., Lapadat R., Webb S., Johnson G.L., Garrington T.P. (2004). MEKK2 regulates the coordinate activation of ERK5 and JNK in response to FGF-2 in fibroblasts. J. Cell. Physiol..

[27] Liu B., Fang M., He Z., Cui D., Jia S., Lin X., Xu X., Zhou T., Liu W. (2015). Hepatitis B virus stimulates G6PD expression through HBx-mediated Nrf2 activation. Cell Death Dis..

[28] Lee H., Kim M.-N., Ryu K.-Y. (2017). Effect of p62/SQSTM1 polyubiquitination on its autophagic adaptor function and cellular survival under oxidative stress induced by arsenite. Biochem. Biophys. Res. Commun..

[29] Cohen-Kaplan V., Livneh I., Avni N., Fabre B., Ziv T., Kwon Y.T., Ciechanover A. (2016). p62-and ubiquitin-dependent stress-induced autophagy of the mammalian 26S proteasome. Proc. Natl. Acad. Sci. USA.

[30] Sanz L., Sanchez P., Lallena M.J., Diaz-Meco M.T., Moscat J. (1999). The interaction of p62 with RIP links the atypical PKCs to NF-κB activation. EMBO J..

[31] Yu H.B., Kielczewska A., Rozek A., Takenaka S., Li Y., Thorson L., Hancock R.E., Guarna M.M., North J.R., Foster L.J. (2009). Sequestosome-1/p62 is the key intracellular target of innate defense regulator peptide. J. Biol. Chem..

[32] Yan X.Y., Zhang Y., Zhang J.J., Zhang L.C., Liu Y.N., Wu Y., Xue Y.N., Lu S.Y., Su J., Sun L.K. (2017). p62/SQSTM1 as an oncotarget mediates cisplatin resistance through activating RIP1-NF-κB pathway in human ovarian Cancer Cells. Cancer Sci..

[33] Teramachi J., Silbermann R., Yang P., Zhao W., Mohammad K.S., Guo J., Anderson J.L., Zhou D., Feng R., Myint K.-Z. (2016). Blocking the ZZ domain of sequestosome1/p62 suppresses myeloma growth and osteoclast formation in vitro and induces dramatic bone formation in myeloma-bearing bones in vivo. Leukemia.

[34] Teramachi J., Myint K.Z.Y., Feng R., Xie X., Windle J.J., Roodman D., Kurihara N. (2011). Blocking the ZZ Domain of Sequestosome 1/p62 suppress the enhancement of myeloma cell growth and osteoclast formation by marrow stromal cells. Blood..

[35] Jiang J., Parameshwaran K., Seibenhener M.L., Kang M.-G., Suppiramaniam V., Huganir R.L., Diaz-Meco M.T., Wooten M.W. (2009). AMPA receptor trafficking and synaptic plasticity require SQSTM1/p62. Hippocampus.

[36] Lamark T., Kirkin V., Dikic I., Johansen T. (2009). NBR1 and p62 as cargo receptors for selective autophagy of ubiquitinated targets. Cell Cycle.

[37] Cha Y.J., Kim H.M., Koo J.S. (2017). Expression of Autophagy-Related Proteins in Hürthle Cell Neoplasm Is Different from That in Follicular Neoplasm. Dis. Mark..

[38] Johansen T., Birgisdottir Å., Huber J., Kniss A., Dötsch V., Kirkin V., Rogov V. (2017). Chapter Nine-Methods for Studying Interactions Between Atg8/LC3/GABARAP and LIR-Containing Proteins. Methods Enzymol..

[39] Maruyama Y., Sou Y.-S., Kageyama S., Takahashi T., Ueno T., Tanaka K., Komatsu M., Ichimura Y. (2014). LC3B is indispensable for selective autophagy of p62 but not basal autophagy. Biochem. Biophys. Res. Commun..

[40] Kirkin V., Lamark T., Sou Y.-S., Bjørkøy G., Nunn J.L., Bruun J.-A., Shvets E., McEwan D.G., Clausen T.H., Wild P. (2009). A role for NBR1 in autophagosomal degradation of ubiquitinated substrates. Mol. Cell.

[41] Lee Y.K., Jun Y.W., Choi H.E., Huh Y.H., Kaang B.K., Jang D.J., Lee J.A. (2017). Development of LC3/GABARAP sensors containing a LIR and a hydrophobic domain to monitor autophagy. EMBO J..

[42] Jiang K., Liu M., Lin G., Mao B., Cheng W., Liu H., Gal J., Zhu H., Yuan Z., Deng W. (2016). Tumor suppressor Spred2 interaction with LC3 promotes autophagosome maturation and induces autophagy-dependent cell death. Oncotarget.

[43] Jain A., Lamark T., Sjøttem E., Larsen K.B., Awuh J.A., Øvervatn A., McMahon M., Hayes J.D., Johansen T. (2010). p62/SQSTM1 is a target gene for transcription factor NRF2 and creates a positive feedback loop by inducing antioxidant response element-driven gene transcription. J. Biol. Chem..

[44] Zhang D.D. (2006). Mechanistic studies of the Nrf2-Keap1 signaling pathway. Drug Metab. Rev..

[45] Ku B.M., Kim D.-S., Kim K.-H., Yoo B.C., Kim S.-H., Gong Y.-D., Kim S.-Y. (2013). Transglutaminase 2 inhibition found to induce p53 mediated apoptosis in renal cell carcinoma. FASEB J..

[46] Ichimura Y., Kumanomidou T., Sou Y.-S., Mizushima T., Ezaki J., Ueno T., Kominami E., Yamane T., Tanaka K., Komatsu M. (2008). Structural basis for sorting mechanism of p62 in selective autophagy. J. Biol. Chem..

[47] Lau A., Wang X.-J., Zhao F., Villeneuve N.F., Wu T., Jiang T., Sun Z., White E., Zhang D.D. (2010). A noncanonical mechanism of Nrf2 activation by autophagy deficiency: direct interaction between Keap1 and p62. Mol. Cell. Biol..

[48] Darvekar S.R., Elvenes J., Brenne H.B., Johansen T., Sjøttem E. (2014). SPBP is a sulforaphane induced transcriptional coactivator of NRF2 regulating expression of the autophagy receptor p62/SQSTM1. PLoS ONE.

[49] Park J.S., Kang D.H., Bae S.H. (2015). PF-4708671, a specific inhibitor of p70 ribosomal S6 kinase 1, activates Nrf2 by promoting p62-dependent autophagic degradation of Keap1. Biochem. Biophys. Res. Commun..

[50] Isogai S., Morimoto D., Arita K., Unzai S., Tenno T., Hasegawa J., Sou Y.-S., Komatsu M., Tanaka K., Shirakawa M. (2011). Crystal structure of the ubiquitin-associated (UBA) domain of p62 and its interaction with ubiquitin. J. Biol. Chem..

[51] Goode A., Layfield R. (2010). Recent advances in understanding the molecular basis of Paget disease of bone. J. Clin. Pathol..

[52] McManus S., Roux S. (2012). The adaptor protein p62/SQSTM1 in osteoclast signaling pathways. J. Mol. Signal..

[53] Laurin N., Brown J.P., Morissette J., Raymond V. (2002). Recurrent mutation of the gene encoding sequestosome 1 (SQSTM1/p62) in Paget disease of bone. Am. J. Hum. Genet..

[54] Hocking L.J., Lucas G.J., Daroszewska A., Mangion J., Olavesen M., Cundy T., Nicholson G.C., Ward L., Bennett S.T., Wuyts W. (2002). Domain-specific mutations in sequestosome 1 (SQSTM1) cause familial and sporadic Paget’s disease. Hum. Mol. Genet..

[55] Durán A., Serrano M., Leitges M., Flores J.M., Picard S., Brown J.P., Moscat J., Diaz-Meco M.T. (2004). The atypical PKC-interacting protein p62 is an important mediator of RANK-activated osteoclastogenesis. Dev. Cell.

[56] Duran A., Linares J.F., Galvez A.S., Wikenheiser K., Flores J.M., Diaz-Meco M.T., Moscat J. (2008). The signaling adaptor p62 is an important NF-κB mediator in tumorigenesis. Cancer Cell.

[57] Zhang P., Zheng Z., Ling L., Yang X., Zhang N., Wang X., Hu M., Xia Y., Ma Y., Yang H. (2017). w09, a novel autophagy enhancer, induces autophagy-dependent cell apoptosis via activation of the EGFR-mediated RAS-RAF1-MAP2K-MAPK1/3 pathway. Autophagy.

[58] Mathew R., Karp C.M., Beaudoin B., Vuong N., Chen G., Chen H.-Y., Bray K., Reddy A., Bhanot G., Gelinas C. (2009). Autophagy suppresses tumorigenesis through elimination of p62. Cell.

[59] Cha-Molstad H., Yu J.E., Feng Z., Lee S.H., Kim J.G., Yang P., Han B., Sung K.W., Yoo Y.D., Hwang J. (2017). p62/SQSTM1/Sequestosome-1 is an N-recognin of the N-end rule pathway which modulates autophagosome biogenesis. Nat. Commun..

[60] Kim P.K., Hailey D.W., Mullen R.T., Lippincott-Schwartz J. (2008). Ubiquitin signals autophagic degradation of cytosolic proteins and peroxisomes. Proc. Natl. Acad. Sci. USA.

[61] Lee Y., Weihl C.C. (2017). Regulation of SQSTM1/p62 via UBA domain ubiquitination and its role in disease. Autophagy.

[62] Lee Y., Chou T.-F., Pittman S.K., Keith A.L., Razani B., Weihl C.C. (2017). Keap1/Cullin3 modulates p62/SQSTM1 activity via UBA domain ubiquitination. Cell Rep..

[63] Komatsu M., Waguri S., Koike M., Sou Y.-S., Ueno T., Hara T., Mizushima N., Iwata J.-I., Ezaki J., Murata S. (2007). Homeostatic levels of p62 control cytoplasmic inclusion body formation in autophagy-deficient mice. Cell.

[64] Itakura E., Mizushima N. (2011). p62 Targeting to the autophagosome formation site requires self-oligomerization but not LC3 binding. J. Cell Biol..

[65] Yang H., Ni H.-M., Guo F., Ding Y., Shi Y.-H., Lahiri P., Fröhlich L.F., Rülicke T., Smole C., Schmidt V.C. (2016). Sequestosome 1/p62 protein is associated with autophagic removal of excess hepatic endoplasmic reticulum in mice. J. Biol. Chem..

[66] Sugiyama M., Yoshizumi T., Yoshida Y., Bekki Y., Matsumoto Y., Yoshiya S., Toshima T., Ikegami T., Itoh S., Harimoto N. (2017). p62 Promotes Amino Acid Sensitivity of mTOR Pathway and Hepatic Differentiation in Adult Liver Stem/Progenitor Cells. J. Cell. Physiol..

[67] Jo E., Li S., Liang Q., Zhang X., Wang H., Herbert T.P., Jenkins T.A., Xu A., Ye J.-M. (2017). Chronic activation of PPARα with fenofibrate reduces autophagic proteins in the liver of mice independent of FGF21. PLoS ONE.

[68] Yang Y., Fukui K., Koike T., Zheng X. (2007). Induction of autophagy in neurite degeneration of mouse superior cervical ganglion neurons. Eur. J. Neurosci..

[69] Boland B., Kumar A., Lee S., Platt F.M., Wegiel J., Yu W.H., Nixon R.A. (2008). Autophagy induction and autophagosome clearance in neurons: relationship to autophagic pathology in Alzheimer’s disease. J. Neurosci..

[70] Li L., Zhang X., Le W. (2008). Altered macroautophagy in the spinal cord of SOD1 mutant mice. Autophagy.

[71] Padman B.S., Nguyen T.N., Lazarou M. (2017). Autophagosome formation and cargo sequestration in the absence of LC3/GABARAPs. Autophagy.

[72] Ravikumar B., Duden R., Rubinsztein D.C. (2002). Aggregate-prone proteins with polyglutamine and polyalanine expansions are degraded by autophagy. Hum. Mol. Genet..

[73] Hanahan D., Weinberg R.A. (2011). Hallmarks of cancer: the next generation. Cell.

[74] Cai-McRae X., Zhong H., Karantza V. (2015). Sequestosome 1/p62 facilitates HER2-induced mammary tumorigenesis through multiple signaling pathways. Oncogene.

[75] Takamura A., Komatsu M., Hara T., Sakamoto A., Kishi C., Waguri S., Eishi Y., Hino O., Tanaka K., Mizushima N. (2011). Autophagy-deficient mice develop multiple liver tumors. Genes. Dev..

[76] Taniguchi K., Yamachika S., He F., Karin M. (2016). p62/SQSTM1—Dr. Jekyll and Mr. Hyde that prevents oxidative stress but promotes liver cancer. FEBS Lett..

[77] Bokun R., Bakotin J., Milasinović D. (1987). Semiquantitative cytochemical estimation of glucose-6-phosphate dehydrogenase activity in benign diseases and carcinoma of the breast. Acta Cytol..

[78] Wang J., Yuan W., Chen Z., Wu S., Chen J., Ge J., Hou F., Chen Z. (2012). Overexpression of G6PD is associated with poor clinical outcome in gastric cancer. Tumor Biol..

[79] Zampella E.J., Bradley E.L., Pretlow T.G. (1982). Glucose-6-phosphate dehydrogenase: A possible clinical indicator for prostatic carcinoma. Cancer.

[80] Aishima S., Fujita N., Mano Y., Iguchi T., Taketomi A., Maehara Y., Oda Y., Tsuneyoshi M. (2010). p62+ Hyaline inclusions in intrahepatic cholangiocarcinoma associated with viral hepatitis or alcoholic liver disease. Am. J. Clin. Pathol..

[81] Xia M., Yu H., Gu S., Xu Y., Su J., Li H., Kang J., Cui M. (2014). p62/SQSTM1 is involved in cisplatin resistance in human ovarian Cancer Cells via the Keap1-Nrf2-ARE system. Int. J. Oncol..

[82] Yu H., Su J., Xu Y., Kang J., Li H., Zhang L., Yi H., Xiang X., Liu F., Sun L. (2011). p62/SQSTM1 involved in cisplatin resistance in human ovarian Cancer Cells by clearing ubiquitinated proteins. Eur. J. Cancer.

[83] Shi C., Pan B.-Q., Shi F., Xie Z.-H., Jiang Y.-Y., Shang L., Zhang Y., Xu X., Cai Y., Hao J.-J. (2018). Sequestosome 1 protects esophageal squamous carcinoma cells from apoptosis via stabilizing SKP2 under serum starvation condition. Oncogene.

[84] Valencia T., Kim J.Y., Abu-Baker S., Moscat-Pardos J., Ahn C.S., Reina-Campos M., Duran A., Castilla E.A., Metallo C.M., Diaz-Meco M.T. (2014). Metabolic reprogramming of stromal fibroblasts through p62-mTORC1 signaling promotes inflammation and tumorigenesis. Cancer Cell.

[85] Wang Y., Zhu W.-G., Zhao Y. (2017). Autophagy substrate SQSTM1/p62 regulates chromatin ubiquitination during the DNA damage response. Autophagy.

[86] Wang Y., Zhang N., Zhang L., Li R., Fu W., Ma K., Li X., Wang L., Wang J., Zhang H. (2016). Autophagy regulates chromatin ubiquitination in DNA damage response through elimination of SQSTM1/p62. Mol. Cell.

[87] Xu L., Li S., Zhou W., Kang Z., Zhang Q., Kamran M., Xu J., Liang D., Wang C., Hou Z. (2017). p62/SQSTM1 enhances breast cancer stem-like properties by stabilizing MYC mRNA. Oncogene.

[88] Leidal A.M., Debnath J. (2014). ‘Doubling down’on the autophagy pathway to suppress tumor growth. Genes. Dev..

[89] Yeo S.K., Wen J., Chen S., Guan J.-L. (2016). Autophagy differentially regulates distinct breast cancer stem-like cells in murine models via EGFR/Stat3 and Tgfβ/Smad signaling. Cancer Res..

[90] Qu X., Sheng J., Shen L., Su J., Xu Y., Xie Q., Wu Y., Zhang X., Sun L. (2017). Autophagy inhibitor chloroquine increases sensitivity to cisplatin in QBC939 cholangiocarcinoma cells by mitochondrial ROS. PLoS ONE.

[91] Fan Z., Yu H., Cui N., Kong X., Liu X., Chang Y., Wu Y., Sun L., Wang G. (2015). ABT737 enhances cholangiocarcinoma sensitivity to cisplatin through regulation of mitochondrial dynamics. Exp. Cell Res..

[92] Zhang A., He W., Shi H., Huang X., Ji G. (2016). Natural compound oblongifolin C inhibits autophagic flux, and induces apoptosis and mitochondrial dysfunction in human cholangiocarcinoma QBC939 cells. Mol. Med. Rep..

[93] He W., Zhang A., Qi L., Na C., Jiang R., Fan Z., Chen J. (2018). FOXO1, a Potential Therapeutic Target, Regulates Autophagic Flux, Oxidative Stress, Mitochondrial Dysfunction, and Apoptosis in Human Cholangiocarcinoma QBC939 Cells. Cell. Physiol. Biochem..

[94] Jin Z., Li Y., Pitti R., Lawrence D., Pham V.C., Lill J.R., Ashkenazi A. (2009). Cullin3-based polyubiquitination and p62-dependent aggregation of caspase-8 mediate extrinsic apoptosis signaling. Cell.

[95] Moscat J., Diaz-Meco M.T., Albert A., Campuzano S. (2006). Cell signaling and function organized by PB1 domain interactions. Mol. Cell.

[96] Moscat J., Diaz-Meco M.T. (2009). p62 at the crossroads of autophagy, apoptosis, and cancer. Cell.

[97] Sooro M.A., Zhang N., Zhang P. (2018). Targeting EGFR-mediated autophagy as a potential strategy for cancer therapy. Int. J. Cancer.

[98] Lu H., Liu Y., Wang D., Wang L., Zhou H., Xu G., Xie L., Wu M., Lin Z., Yu Y. (2016). Galectin-3 regulates metastatic capabilities and chemotherapy sensitivity in epithelial ovarian carcinoma via NF-κB pathway. Tumor Biol..

[99] Gaikwad S.M., Thakur B., Sakpal A., Singh R.K., Ray P. (2015). Differential activation of NF-κB signaling is associated with platinum and taxane resistance in MyD88 deficient epithelial ovarian Cancer Cells. Int. J. Biochem. Cell Biol..

